# Complete response in gallbladder cancer to erlotinib plus gemcitabine does not require mutation of the epidermal growth factor receptor gene: a case report

**DOI:** 10.1186/1471-2407-10-570

**Published:** 2010-10-20

**Authors:** Kabir Mody, Edward Strauss, Robert Lincer, Richard C Frank

**Affiliations:** 1 Department of Medicine, Norwalk Hospital, 34 Maple Street, Norwalk, CT 06856 USA; 2 Department of Radiology, Norwalk Hospital, 34 Maple Street, Norwalk, CT 06856 USA; 3 Department of Surgery, Norwalk Hospital, 34 Maple Street, Norwalk, CT 06856 USA

## Abstract

**Background:**

Gallbladder cancer typically follows an aggressive course, with chemotherapy the standard of care for advanced disease; complete remissions are rarely encountered. The epidermal growth factor receptor (EGFR) is a promising therapeutic target but the activity of single agent oral EGFR tyrosine kinase inhibitors is low. There have been no previous reports of chemotherapy plus an EGFR-tyrosine kinase inhibitor (TKI) to treat gallbladder cancer or correlations of response with the mutation status of the tyrosine kinase domain of the EGFR gene.

**Case presentation:**

A 67 year old man with metastatic gallbladder cancer involving the liver and abdominal lymph nodes was treated with gemcitabine (1000 mg/m2) on day 1 and 8 every 21 days as well as daily erlotinib (100 mg). After four cycles of therapy, the CA 19-9 normalized and a PET/CT showed a complete remission; this response was maintained by the end of 12 cycles of therapy. Gemcitabine was then discontinued and single agent erlotinib was continued as maintenance therapy. The disease remains in good control 18 months after initiation of therapy, including 6 months on maintenance erlotinib. The only grade 3 toxicity was a typical EGFR-related skin rash. Because of the remarkable response to erlotinib plus gemcitabine, we performed tumor genotyping of the EGFR gene for response predicting mutations in exons 18, 19 and 21. This disclosed the wild-type genotype with no mutations found.

**Conclusion:**

This case report demonstrates a patient with stage IV gallbladder cancer who experienced a rarely encountered complete, prolonged response after treatment with an oral EGFR-TKI plus chemotherapy. This response occurred in the absence of an EGFR gene mutation. These observations should inform the design of clinical trials using EGFR-TKIs to treat gallbladder and other biliary tract cancers; such trials should not select patients based on EGFR mutation status.

## Background

Biliary tract cancers (BTC) include carcinomas of the gallbladder and intra- and extra-hepatic bile ducts (cholangiocarcinomas). Gallbladder cancer is the most common type worldwide, affects women more frequently than men and is considered to be the most aggressive form of BTC with the shortest survival [1]. In contrast to cholangiocarcioma, gallbladder cancer (GBC) has a distinct molecular pathogenesis and may require a different therapeutic approach [1,2].

The majority of BTC present at an advanced, incurable stage and are typically treated with chemotherapy drugs such as 5-fluoruracil, gemcitabine and cisplatin, often in combination. Response rates range from approximately 20-40% and median overall survivals from 8-14 months [1]. The most notable advance in the treatment of BTC is the result of a phase III randomized trial of gemcitabine versus gemcitabine plus cisplatin in which the chemotherapy doublet improved overall survival by 3.6 months [3]. In order for further advances to be made, however, it is likely that a targeted biologic therapy will need to be successfully added to chemotherapy, as has become the paradigm in modern oncologic therapy.

The EGFR family is a prominent target of biological therapies against multiple epithelial malignancies. In gastrointestinal carcinomas, monoclonal antibodies targeting EGFR/EGFR-1 (cetuximab, panitumomab) and EGFR-2 (trastuzumab) have become part of the standard treatment armamentaria against colorectal and gastric cancers, respectively [4,5]. In pancreatic cancer, the combination of gemcitabine plus the oral EGFR-tyrosine kinase inhibitor (TKI) erlotinib demonstrated a small but statistically significant improvement in overall survival compared with gemcitabine alone [6]. The data in BTC is much more limited: single agent erlotinib resulted in a 17% progression free survival at 6 months in previously treated patients [7], and both a case report [8] and an ongoing phase II trial [9] support the benefit of adding cetuximab to chemotherapy.

Based on these data and our observation of the activity of erlotinib plus gemcitabine in a patient with refractory gallbladder cancer [10], we utilized this regimen in the front-line setting for the patient herein presented. We also analyzed tumor EGFR DNA for the presence of activating mutations that predict for response to EGFR-TKIs [11]. This analysis is the first published report correlating the EGFR tyrosine kinase domain genotype with response to an EGFR-TKI in a patient with BTC.

## Case Presentation

### Clinical presentation

A 67 year old man in good health presented to our emergency department with right upper quadrant pain. He was a former cigarette smoker with a medical history that included hypertension, atrial fibrillation, and coronary artery disease requiring angioplasty eight years before. His medications included dlitiazem, propafenone, coumadin, aspirin, atenolol, atorvastatin and lisinopril. Physical examination was remarkable only for right upper quadrant tenderness. Routine complete blood count and serum chemistry panel were within normal limits. Ultrasound demonstrated mobile densities in the gallbladder and a submucosal mass. An intended laparoscopic cholecystectomy was converted to an open procedure due to dense fibrosis and inflammation of the gallbladder. The gallbladder specimen revealed a moderately differentiated adenocarcinoma involving the full thickness of the gallbladder wall with extension into the serosa and with both perineural and lymphovascular invasion. One month later, after referral to a tertiary cancer center, he underwent a partial hepatectomy with resection of segments 4 and 5, pelvic lymphadenectomy, common bile duct excision, and reconstruction using a Roux-en-Y hepaticojejunostomy. Pathology revealed residual carcinoma involving the gallbladder bed with extension into the hepatic parenchyma; the lymph nodes were uninvolved. No adjuvant therapy was administered. Fourteen months later, computed tomography (CT) of the abdomen showed new hepatic metastases in segments 5 and 8. Positron emission tomography (PET)/CT scan with 18-fluorodeoxyglucose demonstrated the liver lesions to have SUV 6.3, a focus in the portacaval region of SUV 13.6 and a soft tissue mass inferior to the celiac trunk of SUV 6.0. The CA 19-9 was 48 (upper limit of normal 35 IU/mL) and CEA normal. He remained well with no complaints of abdominal pain, weight loss, or pruritus and the physical examination at the initiation of chemotherapy was unremarkable.

### Treatment and Response

**Figure 1:**
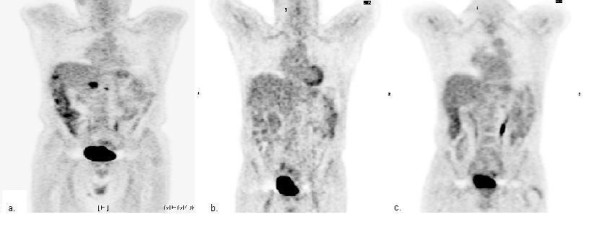
**PET scan images during treatment with erlotinib plus gemcitabine**. Selected PET scan images at (a) diagnosis, (b) after 4 cycles of therapy, and (c) after 12 cycles of therapy, demonstrating a complete response to treatment.

**Figure 2:**
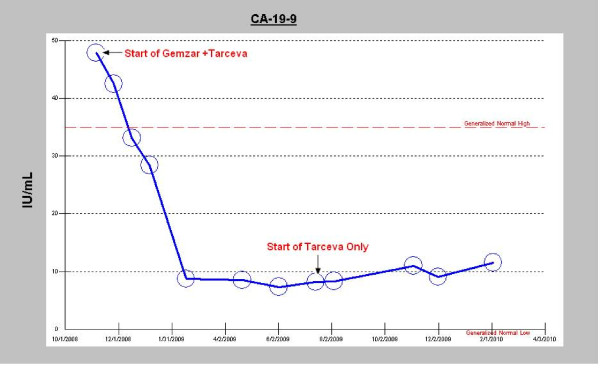
**CA 19-9 levels during treatment**. Graphical depiction of the decline in CA 19-9 tumor marker levels during treatment with erlotinib plus gemcitabine and with maintenance erlotinib.

The patient was treated in a non-protocol fashion with fixed-dose rate gemcitabine (1000 mg/m2 administered at 10 mg/m2 per minute) [12] on day 1 and 8 every 21 days, plus erlotinib 100 mg daily. After four cycles of therapy, a PET/CT scan showed resolution of all FDG-avidity and the CA 19-9 declined into the normal range (Figures 1 and 2). After six cycles of therapy, a CT scan showed no evidence of disease and the patient was given a one month break from gemcitabine (erlotinib was continued) to allow for repair of a large ventral hernia. The patient completed 12 cycles of combination therapy at which point a PET/CT showed a continued complete response of all FDG-avid disease; the CA 19-9 remained at a very low level (Figures 1 and 2).

Given the complete response of the patient's disease to treatment and his desire to take a break from intravenous treatments, further chemotherapy was stopped and he continued single-agent erlotinib. During the six months of maintenance erlotinb, his performance status and quality of life were excellent. The CA 19-9 has slowly increased, though still within the normal range. A follow-up PET/CT after six months of erlotinib showed a localized area of FDG-activity in the portacaval region SUV 9.4, without a clear lymph node or mass seen; all other areas, including the liver, remain without evidence of disease recurrence.

Regarding treatment tolerance, the only grade 3 toxicity (National Cancer Institute Common Terminology Criteria, version 3.0) has been an EGFR-related skin rash on the face, chest and back that has responded well to oral doxycycline and topical clindamycin cream. Minor toxicities have included grade 1 fatigue, grade 2 diarrhea, grade 2 neutropenia and grade 2 anemia.

### EGFR testing

**Figure 3:**
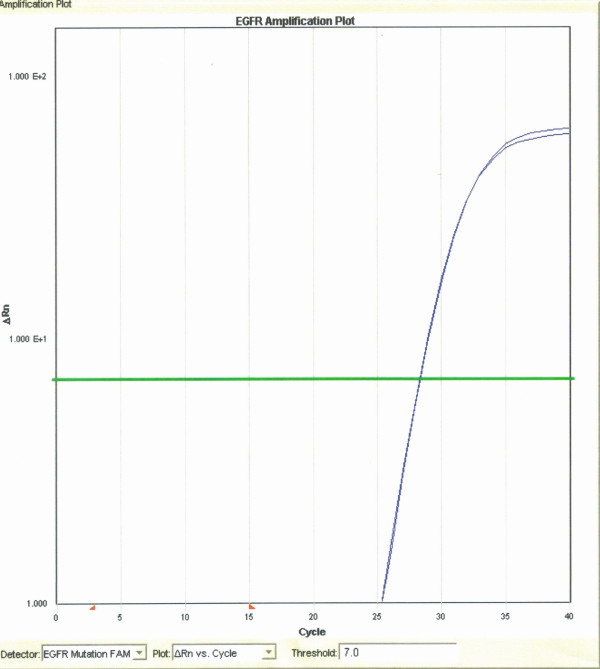
**EGFR mutation analysis**. Polymerase chain reaction amplification plot of exons 18-21 demonstrating the presence of only wild-type EGFR DNA in the patient's tumor sample. (Performed by Clarient Diagnostic Services, Aliso Viejo, CA)

For EGFR mutation analysis, tumor areas of interest were identified, microdissected, and collected cells lysed. After genomic DNA was purified, DNA yield was determined and the sample brought to optimal concentration. Real-time PCR was then used to evaluate for specific mutations, deletions and insertions in the tyrosine kinase domain of the EGFR gene. Eight reactions containing 30 primer and probe sets were used to target specific regions of exons 18-21 as well as the wild-type sequence. EGFR-mutation analysis by real-time PCR for the 29 known mutations, deletions and insertions found in exons 18-21 of the EGFR tyrosine kinase domain revealed only the wild-type sequence (performed by Clarient Diagnostic Services) (Figure 3).

**Figure 4:**
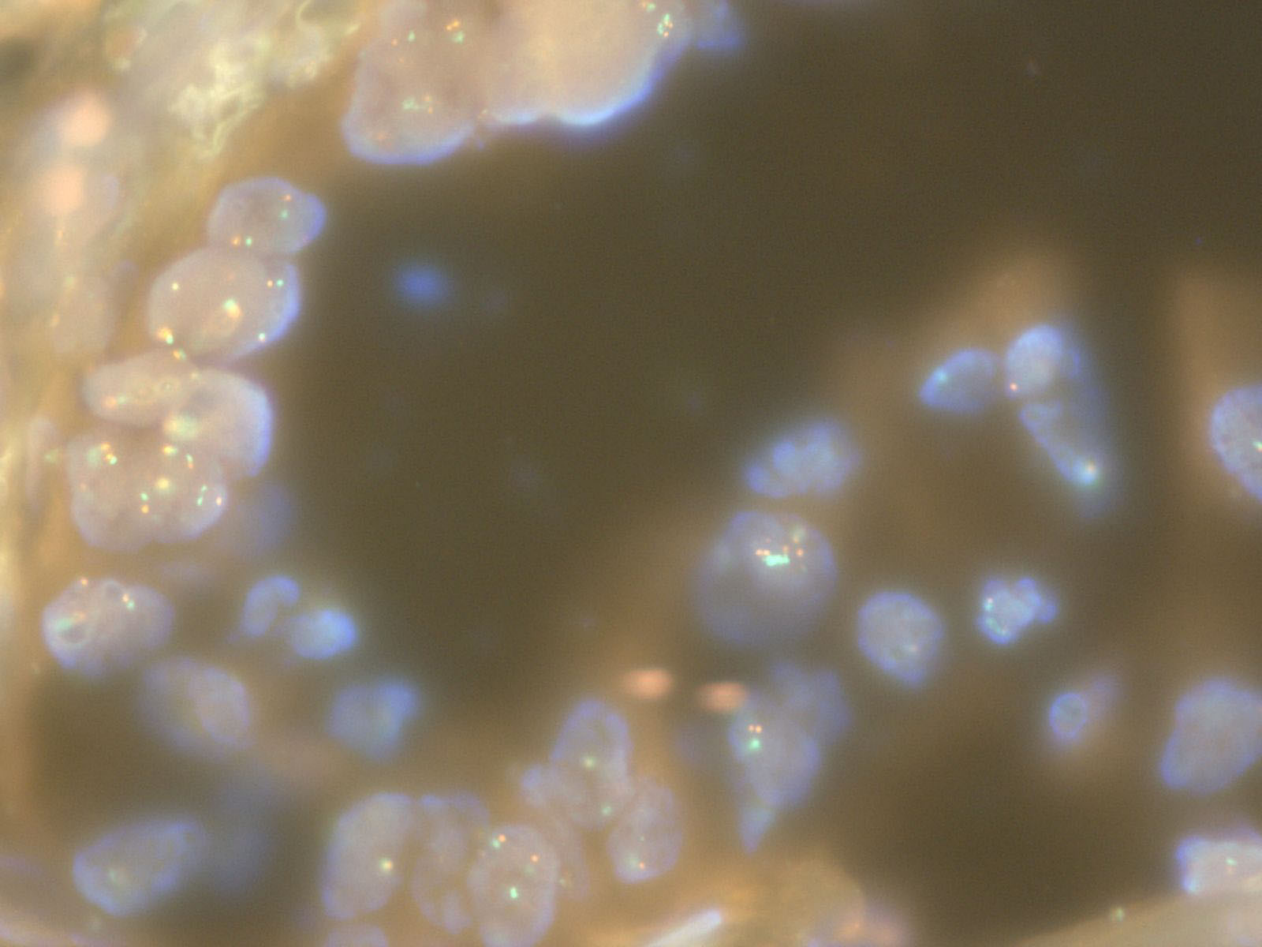
**FISH testing for EGFR amplification**. Fluorescence in-situ hybridization (FISH) testing of the tumor sample using an EGFR-CEP dual color DNA probe (Vysis) demonstrating lack of EGFR gene amplification.

Assessment of EGFR gene amplification was performed by fluorescence in situ hybridization (FISH) testing of the tumor specimen using the EGFR-CEP (chromosome 7 centromere) dual color DNA probe (performed by Genzyme Genetics). This demonstrated that the EGFR gene was not amplified (Figure 4).

## Conclusion

In a recently published landmark phase III trial of cisplatin plus gemcitabine versus gemcitabine alone for the treatment of BTC, no complete responses were seen among 117 gallbladder cancer patients treated [3]. Similarly, in phase II trials of single-agent gemcitabine given to gallbladder [13] and BTC patients [12], no complete responses were observed. In a recent phase II study in BTC of gemcitabine and oxaliplatin plus the angiogenesis-inhibitor bevacizumab, among 35 patients treated there were no complete responses observed by either PET or CT scans, despite an overall response rate of 40% [14].

We took a novel approach to the treatment of our patient with stage IV gallbladder cancer and observed a rarely encountered complete response by both PET/CT and dedicated CT scans. Both the response to erlotinib plus chemotherapy and the prolonged disease control observed with single-agent maintenance erlotinib lead us to conclude that erlotinib plus chemotherapy may be an effective treatment for patients with advanced gallbladder cancer. We can not rule out that the observed response was due solely to treatment with gemcitabine. Yet, our unpublished observations of patients with refractory gallbladder cancer who responded to erlotinib plus chemotherapy and the rarity of complete responses to chemotherapy reported in the literature make this seem unlikely.

The tailored use of EGFR-TKI therapy has been pioneered in the area of lung cancer [15]. Patients with non-small cell lung cancer whose tumors harbor an EGFR mutation have a superior response to TKI therapy compared with chemotherapy alone [16]. In BTC, a 13-15% tyrosine kinase domain mutation rate has been reported and it has been suggested that patients with tumor associated EGFR mutations be preferentially enrolled in clinical trials of EGFR-TKIs [17,18].

The patient with gallbladder cancer presented in this report had a rarely encountered compete response in the absence of a tumor associated EGFR mutation. This would suggest that future studies of EGFR-TKI therapy plus chemotherapy in patients with BTCs should not be restricted to those with EGFR mutations. Since gallbladder cancer and cholangiocarcinoma are distinct clinicopathologic entities (despite being grouped together in most clinical trials), it is possible that erlotinib plus chemotherapy may be more efficacious for patients with gallbladder cancer than those with cholangiocarcinoma. Such hypotheses can only be tested in well-designed clinical trials.

## Consent

Written informed consent was obtained from the patient for publication of this case report and any accompanying images. A copy of the written consent is available for review by the Editor-in-Chief of this journal.

## Abbreviations

Abbreviations have been defined within the manuscript.

## Competing interests

The authors declare that they have no competing interests.

## Authors' contributions

KM drafted the manuscript; aided in acquisition of all data to be included, ES aided in acquisition and interpretation of the data, RL aided in acquisition of the data, RCF made substantial contributions to the conception, acquisition and analysis of the data and editing of this manuscript. All authors have read and approved the final manuscript.

## Authors' Information

KM: Hospitalist, Norwalk Hospital

ES: Radiologist and Radiology Residency Program Director, Norwalk Hospital

RL: Surgeon, Norwalk Hospital

RCF: Oncologist and Director of Research, Whittingham Cancer Center, Norwalk Hospital

## Pre-publication history

The pre-publication history for this paper can be accessed here:

http://www.biomedcentral.com/1471-2407/10/570/prepub
